# Effect of bevel direction on the success rate of ultrasound-guided radial arterial catheterization

**DOI:** 10.1186/s12871-016-0202-5

**Published:** 2016-07-11

**Authors:** Sung-Won Min, Hyong-Rae Cho, Young-Tae Jeon, Ah-Young Oh, Hee-Pyoung Park, Chun Woo Yang, Woo Hee Choi, Byung-Gun Kim

**Affiliations:** 1 Department of Anesthesiology and Pain Medicine, SMG-SNU Boramae Medical Center, Seoul, South Korea; 2 Department of Anesthesiology and Pain Medicine, Myongji Hospital, Goyang, South Korea; 3 Department of Anesthesiology and Pain Medicine, Seoul National University Bundang Hospital, Seongnam, South Korea; 4 Department of Anesthesiology and Pain Medicine, Seoul National University Hospital, Seoul National University College of Medicine, Seoul, South Korea; 5 Department of Anesthesiology and Pain Medicine, Inha University School of Medicine, Inha University Hospital, Sinheung-dong 3-ga, Jung-gu Incheon, 400-711 South Korea

## Abstract

**Background:**

This study assessed the effect of bevel direction on the success rate of ultrasound guided radial artery catheterization.

**Methods:**

A total of 204 patients requiring radial artery catheterization were randomly divided into bevel-up (*n* = 102) and bevel-down (*n* = 102) groups. Success rate, cannulation time, and number of attempts were compared groups.

**Results:**

In the bevel-down group, an arterial line was placed on the first attempt in 86 of 102 (84.3 %; 95 % confidence interval [CI] = 76 % to 90 %) patients versus 73 of 102 (71.6 %; 95 % CI = 62.1 % to 79.4 %) in the bevel-up group (*p* = 0.028). In the bevel-down group, the mean time to a successful radial arterial cannulation was 33.3 ± 6.3 seconds (95 % CI = 32.1-34.6) versus 35.9 ± 7.6 seconds (95 % CI = 34.4-37.2) in the bevel-up group (*p* = 0.011). The median score was 33.2 and interquartile range [IQR] was 10.9 (30.3-41.2) for the mean cannulation time in the bevel-up group. In the bevel-down group, the mean score was 32.3 (IQR 3.90, 30–33.9) for mean cannulation time. In the bevel-down group, 11 of 102 (7 %; 95 % CI = 0 to 16 %) patients developed a posterior wall puncture versus 22 of 102 ((21.6 %; 95 % CI = 14.7 to 17.2 %) in the bevel-up group.

**Conclusion:**

The bevel-down approach during ultrasound-guided radial artery catheterization exhibited a higher success with fewer complications compared to the bevel-up approach.

**Trial registration:**

Clinical Research Information Service is Korean Clinical Trials Registry (KCT0001836). It was registered retrospectively 30th Nov 2015.

## Background

Recent evidence suggests that ultrasound (US)-guided radial artery cannulation can improve the success rate of cannula insertion and decrease the incidence of complications compared to the traditional palpation method [1–3]. US reduce the insertion time required for successful arterial line cannulation [3]. US-guided radial artery catheterization is superior to the palpation method in obese patients, older patients and those with edema, severe hypotension, or vascular anomalies, such as tortuous vessels [4].

Puncture needle placement with US guidance can be performed using either a short-axis (out-of-plane) or a long-axis (in-plane) approach to visualize the needle as it is advanced toward the radial artery [5]. For US-guided radial artery catheterization, the short-axis approach is associated with a lower first-attempt success rate compared to the long-axis method (51 % vs. 76 %) [6].

We hypothesised that using a bevel-down approach with the puncture needle can improve the success of the short-axis approach during US-guided radial artery catheterization. We compared differences between the bevel-down and bevel-up approaches with regard to number of insertion attempts, insertion time, and complication rate.

## Methods

This prospective, single blind, randomized, controlled study was conducted at Seoul National University Bundang Hospital after approval had been obtained from the hospital’s ethics committee (IRB protocol number: B-1201–070–002). Written informed consent include images relating to individual participants was obtained from each participant during a preoperative visit. We explained this is a single-blind study (i.e., subject unaware of group allocation until completion of the study). The study protocol is retrospectively registered with the Korean Clinical Trials Registry (KCT0001711).

Between April 2014 and September 2014, patients between 18 and 80 years of age with an American Society of Anesthesiologists physical status score between I–III and who required radial arterial catheterization for continuous blood pressure monitoring under general anesthesia, were enrolled. Patients with a history of atherosclerosis, hemorrhagic shock, morbid obesity, Raynaud’s disease, or peripheral vascular disease were excluded. Subjects were allocated to either the bevel-up (bevel facing upwards) or bevel-down (bevel facing downwards) group by block randomization. Random numbers generated using a computer-generated randomization table were sealed in an opaque envelope. An operator with experience of more than 100 US-guided radial artery catheterization procedures performed the cannulation.

**Fig. 1:**
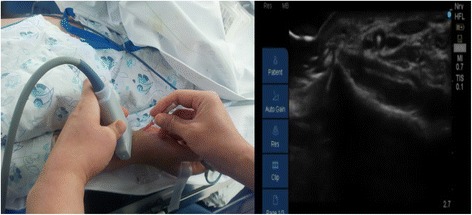
The puncture needle is inserted perpendicular to the transducer (left image) and appears on theultrasound screen (right image) as a dot within the lumen of the radial artery

**Fig. 2:**
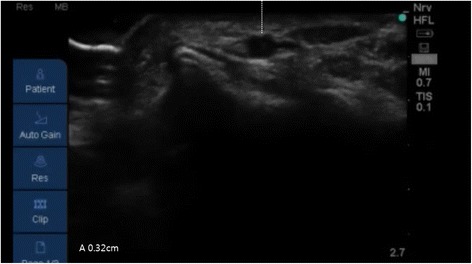
Measurement of the depth from skin of radial artery on the frozen image in ultrasound

**Fig. 3:**
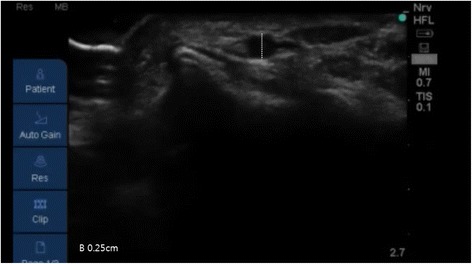
Measurement of the diameter of the radial artery on the frozen image in ultrasound

Following induction of general anesthesia, radial arterial catheterization was performed. With the arm in supination, the wrist was extended over a roll with the hand dorsiflexed at 45°. An observer recorded the time taken (using a stopwatch) by the anesthesiologists to achieve successful US-guided radial artery catheterization. Following antiseptic preparation of the insertion site, an US probe connected to a US system (S-nerve™; Sonosite, Bothell, WA, USA) was used to identify the radial artery in the short-axis view. Using a short-axis approach, a 20-gauge intravenous cannula (BD Angiocath Plus™; Becton Dickinson Medical [S] Pte Ltd, Tuas, Singapore) was placed beneath the center of the transducer at an angle of 30–45°; no local anesthetic was administered prior to catheterization. The puncture needle was inserted with the bevel facing upward in the bevel-up group, and downward in the bevel-down group. When the needle was inserted into a radial artery, an image of its tip appeared on the US screen as a dot within the lumen of the vessel (Fig. 1). Entry of the needle into the artery was indicated by adequate return of free-flowing arterial blood. The diameter (Fig. 2) and depth of the artery (Fig. 3) (the latter defined as the distance from the skin to the outer wall of the artery) were measured from the US image.

Insertion time, defined as the time between the US probe contacting the skin and the placing of the catheter into the radial artery and the number of attempts, were recorded. The ease of insertion was indexed by the success rate on the first attempt. Total success rate, first attempt success rate, second attempt success rate, third attempt success rate, and failure rate in both groups were recorded. Failure of cannulation was defined as more than three attempts at cannulation, because of the possibility for numerous attempts. New attempt was defined as a new penetration of the skin with the needle, followed by an unlimited number of redirections under the skin, as needed. A new catheter was required for new attempt.

**Fig. 4:**
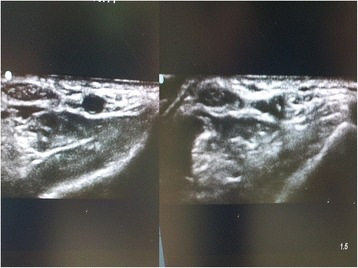
Image of a posterior wall hematoma. Left, pre-catheterization right, post-catheterization

Complications including thrombosis, hematoma, vasospasm and posterior wall damage were recorded by an observer. Complications was monitored and detected on US image clinically during and after radial arterial cannulation by an observer (Fig. 4).

### Statistical analysis

SPSS for Windows software package ver. 19.0 (SPSS, Inc., Chicago, IL, USA) was used for statistical analysis. Data are presented as mean ± SD or median (range) as appropriate. The categorical end point variables were analyzed using the *χ*2 test, or Fisher exact test if the subject count in any contingency table cell was expected to be <5. Differences with *P* value <0.05 and 95 % CIs excluding 0 were considered statistically significant. Student’s *t*-test was used for group comparison of age, height, weight, and radial artery diameter and depth from the skin. A *p*-value of 0.05 was taken to indicate statistical significance.

### Sample size analysis

The first success rate for US-guided radial artery catheterization using the short-axis approach is reportedly 51 % [6]. Based on this report, we estimated that 92 patients would provide 80 % power for detecting a 50 % improvement in the success rate, from 60 % to 90 % at a 2-tailed α risk of 0.05. Allowing for dropouts and technical problems, 204 patients were enrolled into the study.

## Results

**Fig. 5:**
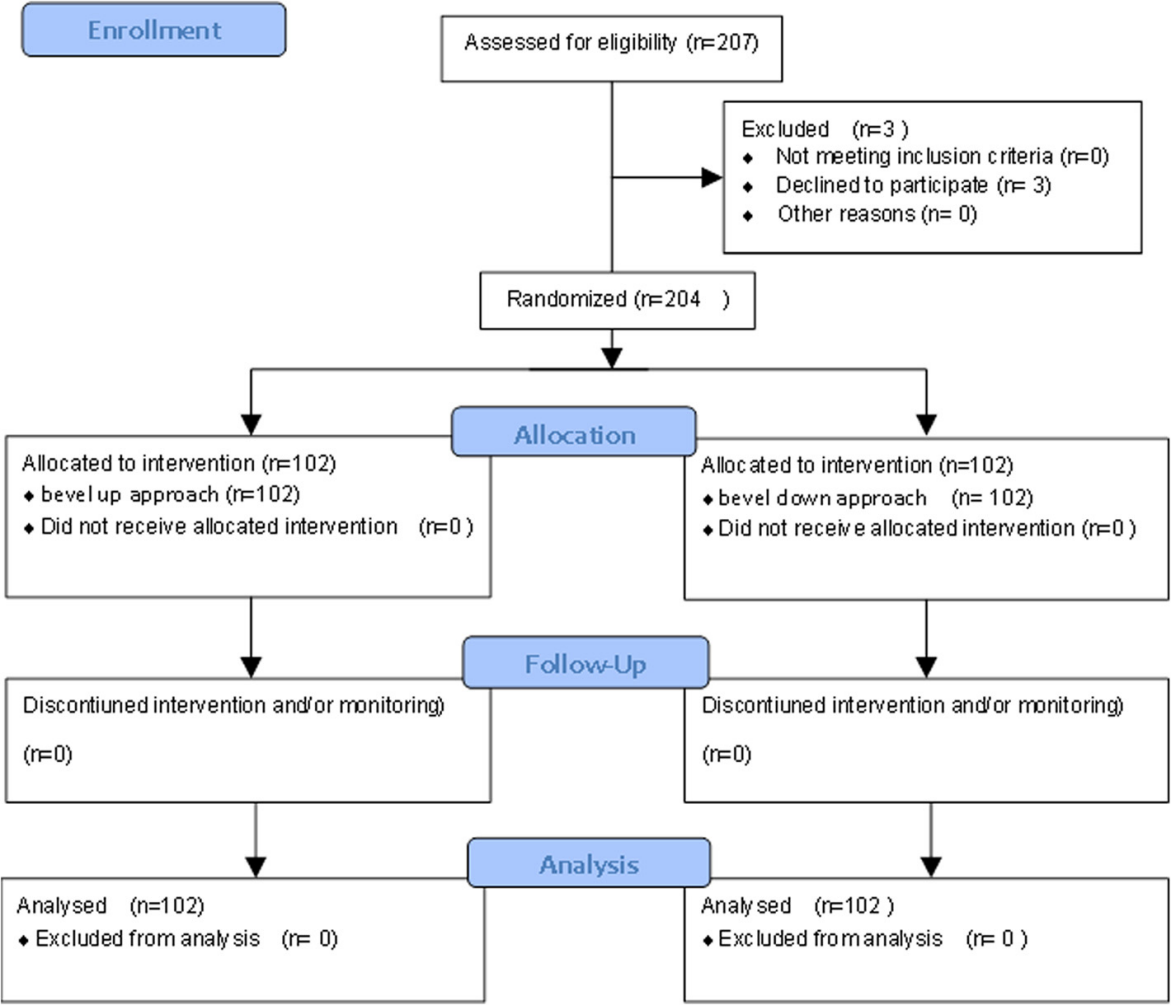
Patient randomization and follow-up according to the CONSORT (Consolidated Standards of Reporting Trials) guidelines

A total of 204 patients were enrolled over a 5-month period, with 102 randomized to the bevel-up group and 102 to the bevel-down group. Of the 207 patients enrolled, three were subsequently excluded when they declined to participate. A total of 204 patients were finally included (Fig. 5).

The two groups were comparable in terms of demographic data and depth and diameter of the radial artery (Table 1). The two groups did not differ significantly in demographic data and depth and diameter of the radial artery. None of the patients in the both group required more than four attempts.

**Table 1:** Patient characteristics

	Bevel-up group (*n* =102)	Bevel-down group (*n* = 102)
Sex, male/female	45/57	46/56
Age (years)	62 ± 13	61 ± 12
Height (cm)	161 ± 9	160 ± 9
Weight (kg)	63 ± 10	63 ± 10
Diameter of artery (mm)	2.4 ± 0.6	2.5 ± 0.5
Depth from skin (mm)	2.5 ± 0.3	2.5 ± 0.2

In the bevel-down group, an arterial line was placed on the first attempt in 86 of 102 (84.3 %; 95 % confidence interval [CI] = 76 % to 90 %) patients versus 73 of 102 (71.6 %; 95 % CI = 62.1 % to 79.4 %) in the bevel-up group (*p* = 0.028). In the bevel-down group, the mean time to a successful radial arterial cannulation was 33.3 ± 6.3 seconds (95 % CI = 32.1-34.6) versus 35.9 ± 7.6 seconds (95 % CI = 34.4-37.2) in the bevel-up group (*p* = 0.011). The median score was 33.2 and interquartile range [IQR] was 10.9 (30.3-41.2) for the mean cannulation time in the bevel-up group. In the bevel-down group, the mean score was 32.3 (IQR 3.90, 30–33.9) for mean cannulation time. In the bevel-down group, 11 of 102 (7 %; 95 % CI = 0 to 16 %) patients developed a posterior wall puncture versus 22 of 102 ((21.6 %; 95 % CI = 14.7 to 17.2 %) in the bevel-up group. (Table 2). Complications such as edema, thrombosis, and vasospasm were not observed in either group.

**Table 2:** Catheterization related variables

Parameter	Bevel-Up group (*n* =102)	Bevel-down group (*n* = 102)	95 % CI of mean	Odds ratio	*P* value
Attempts					
First attempt	73 (71.6 %)	86 (84.3 %)	0.6216-0.7642/	2.14	0.028
			0.7603-0.9011		
Second attempt	23 (22.5 %)	14 (13.7 %)	0.1552-0.3157/	1.83	0.103
			0.0836-0.2173		
Third attempt	6 (5.9 %)	2 (2.0 %)	0.02720.1224/	3.13	0.151
			0.0054-0.0687		
overall	102 (100 %)	102 (100 %)			1.00
Mean cannulation time(sec)	35.9 ± 7.6	33.3 ± 6.3	34.4-37.4/		0.011
			32.1-34.6		
Posterior wall hematoma	22 (21.6 %)	11(10.8 %)	−0.0009 - 0.215	2.28	0 .0365

## Discussion

US-guided radial artery cannulation under the short-axis view was more successful on the first attempt using the bevel-down approach, which was also associated with reduced insertion time and posterior wall puncture. The effect of bevel direction under US-guided vascular access has not previously been investigated during radial artery catheterization. The bevel-down approach is associated with reduced posterior wall hematoma during internal jugular vein catheterization compared to the bevel-up approach [7]. The incidence of posterior wall puncture using the short-axis approach is reportedly between 31 % and 56 % [6, 8] although in our study the incidence was lower. It appears that the bevel-down approach could facilitate the avoidance of posterior radial arterial wall injury during US-guided radial arterial catheterization. We suspect that a similar mechanism might be involved in arterial line cannulation. The average diameter of the radial artery is approximately 2.5 mm, while the bevel length at the distal end of the needle is 1.1 mm, with the needle tip cut obliquely at an acute angle. There appears to be insufficient space between the needle tip and posterior vessel wall. We speculate that sharp tips may be more likely to transfix the artery using the bevel-up approach.

The long-axis approach affords improved visualization of the needle tip at the time of puncture [5]. It is often difficult to maintain visualization of the needle tip when performing a procedure using the short axis [5]. However, the short-axis approach, which was used in the present study, benefits from the fact that novice users can locate the vessel more easily and achieve vascular access more rapidly compared to the long-axis approach [2]. Users also rated the short-axis approach as being more straightforward, although not significantly. Several measures to increase the success rate of the short-axis approach have been suggested. These include using a visible marker (i.e., a suture on the midpoint of the US probe) [9]. Subcutaneous injection of saline also improves the success rate of the short-axis method in pediatric patients [10]. However, such measures require additional interventions. We suggest that merely changing the bevel direction might improve the success rate and reduce the incidence of posterior wall hematoma. Moreover, our method can be applied to already-established techniques. Accidental hematoma of the posterior wall of the radial artery may occur due to other factors such as the ability of the operator, characteristics of ultrasound equipment, distance from needle entry to transducer, or other factors [5]. Further studies are necessary to prospectively compare complication rates, particularly posterior radial artery wall penetration, of both approaches during US-guided radial arterial catheterization.

A limitation of our study was that the operator could not be blinded to the type of cannulation technique, which represents a possible source of bias. Still another potential limitation was that cannulation was performed by an experienced operator; results achieved might not be applicable to novice users. A third limitation was not including challenging cases, such as peripheral vascular disease patients due to concern of radial arterial catheterization related vascular infarct and thrombosis. All of these might prevent full generalization of our results. We also excluded the more difficult to catheterize patients such as those who were obese. Patients in shock or with low blood pressure were not included.

A larger study population may be needed to demonstrate the benefit of using the bevel down approach during US-guided radial arterial catheterization for challenging cases.

## Conclusion

We conclude that the use of the bevel-down approach during US-guided radial artery cannulation under a short-axis view improves the first-attempt success rate compared to the bevel-up approach. The bevel-down technique is associated with reduced insertion time and posterior wall hematoma.

## Abbreviations

ASA, American society of anesthesiologist; BMI, body mass index; CI, confidence interval; IQR, interquartile range; US, ultrasound
